# SOCIOECONOMIC DETERMINANTS OF FRAILTY TRAJECTORIES AMONG OLDER ADULTS IN SWITZERLAND (2004–2020)

**DOI:** 10.1093/geroni/igad104.3044

**Published:** 2023-12-21

**Authors:** Mengling Cheng

**Affiliations:** Swiss Centre of Expertise in Life Course Research, Lausanne, Vaud, Switzerland

## Abstract

Frailty in old age is a major public health challenge. Frail older adults are at higher risk of adverse health outcomes and in greater needs for health services as compared to non-frail older adults. An emerging perspective views frailty as a dynamic process and older adults are likely to transition to improved frailty status. However, little is known about the socioeconomic determinants of frailty trajectories over the later life course. This study investigated how frailty evolves and transitions over time and how socioeconomic status impacts frailty trajectories. Data from the Survey of Health, Ageing and Retirement in Europe 2004-2020 (N=4,621 adults aged 50-105 in Switzerland) were analyzed. Fried’s phenotype was used to operationalize frailty. Changes in frailty status at a 2-year interval were used to identify frailty trajectories. Growth curve models were performed to investigate how age, sex, education, occupation, and income determined frailty trajectories over the later life course. This study had three findings. First, three frailty states were identified: non-frail, pre-frail, and frail. Second, five frailty trajectories were identified: stable frail, worsening, mixed, improving, and stable non-frail. Third, individuals aged 65 and above, women, and socioeconomically disadvantaged individuals had a higher risk of being stable frail and having a worsening frailty trajectory. Findings indicate that frailty intervention programs in Switzerland need to prioritize individuals in middle age and old age, women, and individuals with lower socioeconomic status to tackle the challenge of frailty.

